# The Hospital

**Published:** 1917-10-27

**Authors:** 


THE
### INSTITUTIONAL WORKER
Being a Special Supplement to "The Hospital"

### OUR BUREAU OF INFORMATION.

### Rules for Correspondents.

1. Every letter must be accompanied by the coupon to be cut from the back cover (inside page) of THE HOSPITAL, current issue, and must contain the name and address of the correspondent with pseudonym for publication if desired. Replies by post cannot be given save under exceptional circumstances at the Editor's discretion.

2. Letters from Approved Homes in reply to special needs published in the Bureau should state terms and full particulars, and be sent prepaid under cover to the Editor of the Bureau with name written across coupon for identification.

3. Proprietors of Homes which have not yet been entered on the List of Approved Homes, but have spare accommodation likely to suit special needs, are invited to write for an application form for registration. The fee for registration, which includes two announcements of the Home in the Bureau and other privileges, is 10s.

4. All communications to be addressed to the Editor of THE HOSPITAL, 28 Southampton Street, Strand, London, W.C. 2, and marked "Bureau of Information."

### INSTITUTION FACTS AND FIGURES.

### Starting a Venereal Block.

#### Answer to September Question.

By M. B. M.—(*Continued from October* 20.)

THE question was:

**It is proposed to start a venereal block, containing two wards of twelve beds each (male and female), a clinic, and an out-patients' department. (a) What nursing staff will be required, and how should it be distributed? (b) What equipment will be necessary for each department?**

#### II. Equipment.

WARDS.—The usual ward furniture would be required in the wards—*i.e.*, bedsteads, lockers, chairs, tables, doctor's table, dressing-tables, screens, etc. One or two points about the ward furniture which might have special attention are the electric-light cords. Instead of being covered with the usual silk, it would be better to have them of rubber, so that they could be easily cleaned with a disinfectant. The lockers should be so arranged that the backs slide out to enable them to be scrubbed right through at the end of each patient's treatment. If there are any easy-chairs, they should be covered with American cloth, not cretonne or absorbent material.

CLINIC AND OUT-PATIENTS.—The physician's room would be fitted with chairs, special table, steriliser, washing sinks, etc.

The necessary equipment for giving douches would be arranged in one of the rooms. Beyond this there is only the ordinary furniture needed in every out-patient department.

LABORATORY.—This would be somewhere near the ward. It would be fitted with a steriliser for needles, instruments, etc., urine test cupboard, specimen glasses, a desk, and a place to keep the records of the cases.

BATH-ROOM.—The bath should be made of porcelain so that it can be easily kept clean. It would be nice to have hot pipes fitted here, so as to dry towels, etc.

SINK-ROOM.—Besides the usual sink-room arrangements there should be a steriliser large enough to take the bed-pans and bowls, which should be sterilised after each patient before being used by another one.

LAVATORIES.—There should be as little wood used for the seats as possible, as excretions are apt to soak in. It is better to leave the seats unvarnished, so that they can be thoroughly scrubbed.

A separate lavatory under lock and key should be provided for the nursing staff.

WARD KITCHEN OR SCULLERY.—Besides the ordinary sinks, cupboards, etc., as used in sculleries attached to wards, there should be a large steriliser for the crockery. It would be necessary to sterilise the used crockery between each meal; or if each patient kept his or her own crockery, it would only require doing when this patient was discharged.

The average stay of each syphilitic patient would be twenty-four hours, unless the patient had a "reaction," and in this case the stay would be longer.

CROCKERY.

It would be advisable to have all the crockery and utensils used in this block of a different pattern from the rest of the crockery used in the hospital, so that if it strayed to any other part it would be detected at once.

LINEN.

A larger supply of linen will be required than in an ordinary ward, as the change of patients will be much more frequent as compared with other wards. It would not be possible to state the amount per bed unless the laundry arrangements were known. The linen used in the whole of this department should be marked with a distinct mark, but not so distinct as to draw attention to it except by those concerned. For instance, the night-gowns might have a blue collar, the quilts blue check, towels a blue border, dressing-gowns should be made of washing material of special pattern, and so on. This would enable the head of the laundry to see that this linen did not stray amongst the other linen before being disinfected; but after being disinfected it could safely go through with the other linen, and at the same time the attention of the laundry workers would not be drawn to it.

The soiled linen should be taken to the laundry in zinc pails, which can be easily kept clean and disinfected.

### X-Ray Films.

IT is advantageous for several reasons to use flexible *x*-ray films instead of glass plates. In the first place they are unbreakable; glass plates are frequently broken when taking radiograms and in course of transmission from one institution to another. Films being flexible, they can be adapted to a contracted joint and more detail of the structure and pathological changes can be recorded than would be possible if a rigid glass plate were used. A full lateral view of the cervical column can be obtained by adjusting the film between the head and shoulder and arranging the tube so as to avoid undue distortion of the shadow. Austin Edwards, Ltd., of Warwick, prepare a double-coated film which gives results comparable with the best plates in the market. These films are coated on both sides, and that side which is placed next to the patient can be distinguished by a mark in the corner. The process of development, fixing, and washing is quite simple, the films remaining perfectly flat. Each film has two holes by which it can be suspended when fixing, washing, and drying. Another advantage is that, the films being thin, two or more radiographs of the same part can be taken at one exposure. In the case of possible chemical defects, the value of this is obvious; further, without involving additional labour, two records can be obtained, thus saving the trouble of printing and avoiding loss of detail in reproduction.—RADIOGRAPHER.

### QUESTION for OCTOBER.

### AN ISOLATION HOSPITAL DIFFICULTY.

In an isolation hospital wards are often closed for months at a time. In spite of good ventilation and dry weather, the enamel of the bedsteads peels off, and the stoves get rusty very quickly. Explain fully what can be done to prevent this.

RULES.

The following rules must be observed:—

1. Contributions must be written on one side of the paper. Brevity and terseness are desirable features. The MSS. must bear the name and address of the sender and be accompanied by coupon to be cut from the back cover (inside page) of the current issue of The Hospital. A pseudonym must be chosen if the name is not to be published.

2. Contributions must be addressed to the Editor of The Hospital, Institutional Worker Supplement, 28 & 29 Southampton Street, Strand, London, W.C. 2, should reach him before the end of the current month, and be marked in the left-hand corner "Facts and Figures."

**A minimum payment of Half-a-Guinea will be made for each published answer.**

### QUESTIONS INVITED.

In connection with our Question Box, we offer every month five shillings each for the two best questions which are sent in for consideration. The questions may be on any institutional subject and concern any department, from the secretary's to the porter's, or the matron's to the domestic staff; the one essential is that they must be practical and deal with points that have a definite relation to hospital or institutional administration, upkeep, finance, or management. Points relating to artificers' work, housekeeping, the laundry, out-patients, and other practical matters will be welcome. It is hoped that thus, when difficult or doubtful points arise in the course of their work, institutional workers will be encouraged to put them in the form of a question and send them to the Editor, The Hospital Bureau of Information, 28 Southampton Street, Strand, W.C. 2, marked "Question Box."

The best questions will be published in due course and our readers will be given the opportunity of answering them. Thus all institutional workers may be able to co-operate with the view of helping the work and smoothing the difficulties of each other. In short, we want the Question Box of the *Institutional Worker* to be freely used by every worker habitually.

### ENQUIRIES AND ANSWERS.

### *EMPLOYMENT AND TRAINING.*

### Training for Health Visitors.

We suggest that you study either under the direction of the Royal Sanitary Institute, 90 Buckingham Palace Road, S.W. 1, or the National Health Society, 53 Berners Street, W. 1. Write to the respective secretaries for the necessary information. There are many vacancies for health visitors advertised in the nursing journals from time to time.—Welfare.

### *THE HOUSEKEEPERS' DEPARTMENT.*

### The Value of Dried Fruits.

The food value of dried fruits is considered to be high. The principal nutritive element in dried fruits is usually sugar, which is the most easily digested of all the carbohydrates. Raisins, prunes, and apples have a high percentage of sugar, and as sugar is one of the most important factors in producing muscular energy for the body, the nutritive value of dried fruits must be appreciated. Figs, apricots, and dates are also wholesome food.—Interested.

### Steamed Custard for Invalids.

For a small steamed custard you will require a quarter of a pint of milk, one egg and an extra yolk, one teaspoonful of castor sugar, and vanilla to taste. Beat the eggs well together, but do not froth them; bring the milk to boiling-point, then, when it has cooled a little, pour it gradually on to the eggs, stirring well all the time. Add the sugar and a few drops of vanilla (if liked); well butter some small moulds or cups, and strain in the custard. Place a piece of buttered paper over each mould and put them in a saucepan with boiling water to come barely half-way up, put the lid on the pan, and steam the custards very slowly until they are quite firm. If they are cooked quickly they will be full of holes and watery, also be sure that the milk is not *boiling* when poured over the eggs, otherwise the eggs will curdle and the custard will be spoilt. The custard will turn out of the mould better if it is allowed to stand two or three minutes after being lifted out of the pan.—Private Nurse.

### *MISCELLANEOUS.*

### Nurses' Missionary League.

The autumn reunion of the Nurses' Missionary League will be held in University Hall, Gordon Square, London, W.C., on Wednesday, October 31, 1917. The morning session opens at 10.30, the afternoon conversazione at 2.30, and the evening session at 6.45. Communicate with Miss Richardson, Sloane Gardens House, 52 Lower Sloane Street, S.W. 1, who will give you the information you require.—C. E.

### Preparation for the Treatment of Burns.

The preparation you refer to is probably amberine. It is frequently used in hospitals for the treatment of burns, apparently with marked success. The wax-like cake when melted down is applied with a brush; this can, however, be more efficiently and economically done by the use of a special spray. Both the material and the spray may be purchased from the Anglo-French Drug Company, Ltd., Gamage Buildings, Holborn.—Sister A.

### Economy in Paint.

You will find it ultimately somewhat costly to paint the walls of your hospital the same colour from the frieze to the floor, for whereas the upper part will remain in good condition for a considerable period, the lower part, on account of the hard usage it gets, requires repainting more frequently. You should divide the walls or stencil band shoulder high, painting the lower part a darker colour. When this is scratched and worn it can have a fresh coat of paint, and the upper part need only be washed.—Economist.

### Destruction of Rats.

This subject has been referred to on several occasions in these columns. It is practically impossible entirely to rid old premises of rates and mice unless the cause be removed, and it is not always possible to do so. They may be attracted by an adjoining stable or premises where food of any description is stored. It is possible to poison the rats without creating a nuisance through the decomposition of dead bodies. The matter is best dealt with by experts, and we advise you to enter into a contract with a firm which undertakes work of this sort. Write us again if you want further information. —Heather.

### Ratio of Patients and Staff.

The number of staff employed at a hospital in relation to the number of patients depends upon the character of the institution; upon the construction of the building, the facilities of administration, and the amount of departmental work done in the hospital. For instance, the number of staff must be higher in relation to the number of patients at a hospital which runs its own laundry, its pathological department, and so on, than at a hospital which pays to have the work done. The proportion of staff to patients may be as low as and even lower than one to two, and in some institutions it may be as high as two to one. It must be borne in mind that no hospital that is under-staffed can give efficient service. The fact is sometimes overlooked, when discussing bare figures, that provision has to be made for night as well as for day duty.—Harassed.

